# Canonical and Novel Non-Canonical Cholinergic Agonists Inhibit ATP-Induced Release of Monocytic Interleukin-1β via Different Combinations of Nicotinic Acetylcholine Receptor Subunits α7, α9 and α10

**DOI:** 10.3389/fncel.2017.00189

**Published:** 2017-07-05

**Authors:** Anna Zakrzewicz, Katrin Richter, Alisa Agné, Sigrid Wilker, Kathrin Siebers, Bijan Fink, Gabriela Krasteva-Christ, Mike Althaus, Winfried Padberg, Arik J. Hone, J. Michael McIntosh, Veronika Grau

**Affiliations:** ^1^Laboratory of Experimental Surgery, Department of General and Thoracic Surgery, Justus-Liebig-University GiessenGiessen, Germany; ^2^Institute of Anatomy and Cell Biology, Justus-Liebig-University GiessenGiessen, Germany; ^3^Institute of Anatomy and Cell Biology, Saarland UniversityHomburg, Germany; ^4^Member of the German Centre for Lung ResearchGiessen, Germany; ^5^Institute of Animal Physiology, Justus-Liebig-University GiessenGiessen, Germany; ^6^School of Biology, Newcastle UniversityNewcastle upon Tyne, United Kingdom; ^7^Department of Biology, University of UtahSalt Lake City, UT, United States; ^8^George E. Wahlen Veterans Affairs Medical CenterSalt Lake City, UT, United States; ^9^Department of Psychiatry, University of UtahSalt Lake City, UT, United States

**Keywords:** acetylcholine, *CHRNA*, glycerophosphocholine, inflammasome, interleukin-1beta, lysophosphatidylcholine, nicotine and phosphocholine

## Abstract

Recently, we discovered a cholinergic mechanism that inhibits the adenosine triphosphate (ATP)-dependent release of interleukin-1β (IL-1β) by human monocytes via nicotinic acetylcholine receptors (nAChRs) composed of α7, α9 and/or α10 subunits. Furthermore, we identified phosphocholine (PC) and dipalmitoylphosphatidylcholine (DPPC) as novel nicotinic agonists that elicit metabotropic activity at monocytic nAChR. Interestingly, PC does not provoke ion channel responses at conventional nAChRs composed of subunits α9 and α10. The purpose of this study is to determine the composition of nAChRs necessary for nicotinic signaling in monocytic cells and to test the hypothesis that common metabolites of phosphatidylcholines, lysophosphatidylcholine (LPC) and glycerophosphocholine (G-PC), function as nAChR agonists. In peripheral blood mononuclear cells from nAChR gene-deficient mice, we demonstrated that inhibition of ATP-dependent release of IL-1β by acetylcholine (ACh), nicotine and PC depends on subunits α7, α9 and α10. Using a panel of nAChR antagonists and siRNA technology, we confirmed the involvement of these subunits in the control of IL-1β release in the human monocytic cell line U937. Furthermore, we showed that LPC (C16:0) and G-PC efficiently inhibit ATP-dependent release of IL-1β. Of note, the inhibitory effects mediated by LPC and G-PC depend on nAChR subunits α9 and α10, but only to a small degree on α7. In *Xenopus* *laevis* oocytes heterologously expressing different combinations of human α7, α9 or α10 subunits, ACh induced canonical ion channel activity, whereas LPC, G-PC and PC did not. In conclusion, we demonstrate that canonical nicotinic agonists and PC elicit metabotropic nAChR activity in monocytes via interaction of nAChR subunits α7, α9 and α10. For the metabotropic signaling of LPC and G-PC, nAChR subunits α9 and α10 are needed, whereas α7 is virtually dispensable. Furthermore, molecules bearing a PC group in general seem to regulate immune functions without perturbing canonical ion channel functions of nAChR.

## Introduction

Interleukin-1β (IL-1β) is a potent pro-inflammatory cytokine of innate immunity that importantly contributes to host defense against pathogens (Vladimer et al., 2013). Production and secretion of IL-1β are strictly controlled and typically require two independent danger signals (Rathinam et al., 2012). The presence of potential pathogens is commonly sensed by Toll-like receptors that lead to cytoplasmic expression of pro-IL-1β. A prototypical second signal is extracellular adenosine triphosphate (ATP) originating from the cytoplasm of damaged host cells (Trautmann, 2009). ATP binds to the ionotropic P2X7 receptor, which enables efflux of potassium ions. This induces the assembly of the NLRP3 (NLR family, pyrin domain containing protein 3) inflammasome and activation of the protease caspase-1, which eventually cleaves pro-IL-1β and enables the release of mature bioactive IL-1β (Ferrari et al., 2006; Gross et al., 2011; Ozaki et al., 2015). Excessive systemic release of IL-1β can be dangerous for the host (Dinarello et al., 2012). Elevated systemic levels of IL-1β can cause fever, shock, systemic inflammatory response syndrome (SIRS) and can lead to life-threatening multi-organ damage (Cauwels et al., 2014). Mechanisms controlling IL-1β maturation are of substantial clinical interest but so far have remained largely unexplored.

Recently, we demonstrated that activation of nicotinic acetylcholine (ACh) receptors (nAChRs) by classical ligands like ACh, nicotine (Nic) and choline (Cho) inhibits ATP signaling (Hecker et al., 2009, 2015; Mikulski et al., 2010; Richter et al., 2016), activation of caspase-1 and release of IL-1β by human monocytes (Hecker et al., 2015; Richter et al., 2016). Interestingly, activated monocytes, T cells and vascular endothelial cells can endogenously produce ACh, which presumably acts in an autocrine or paracrine fashion (Hecker et al., 2009; Wilczynska et al., 2011; Kawashima et al., 2012). In addition to conventional nAChR agonists, we demonstrated that free phosphocholine (PC), a common metabolite of phosphatidylcholine and PC-modified macromolecules can also effectively inhibit the ATP-dependent release of IL-1β by human and murine monocytes (Hecker et al., 2015; Richter et al., 2016). Gene-silencing experiments and pharmacological studies demonstrated that nAChR subunits α7, α9 and/or α10 are involved in the cholinergic inhibition of IL-1β release (Hecker et al., 2015). The nAChR subunits α9 and α10 contribute to the inhibitory effect of PC (Richter et al., 2016). It is however unclear, if the effect of PC also depends on subunit α7 and if receptor subunits α7, α9 and α10 cooperate in monocytic cells or exert redundant functions. Similarly, dipalmitoylphosphatidylcholine (DPPC), the main lipid component of pulmonary surfactant, inhibits ATP-driven inflammasome activation in monocytes. DPPC signals via the nAChR subunit α9 in combination with either subunit α7 or α10 (Backhaus et al., 2017).

Phosphatidylcholines possess a PC head group and two non-polar fatty acid chains. Enzymatic removal of one fatty acid results in lysophosphatidylcholine (LPC), which can be further metabolized to glycerophosphocholine (G-PC). LPC has been shown to modulate responses of the innate and adaptive immune system by various non-cholinergic mechanisms (Kabarowski et al., 2002; Stock et al., 2006; Carneiro et al., 2013). The potential involvement of LPC and G-PC, two molecules bearing a PC head group, in the cholinergic regulation of ATP-mediated IL-1β release by monocytes has not yet been tested.

The purpose of this study was to investigate if nAChR subunits α7, α9 and α10 interact in monocytes and which nAChR subunit composition is necessary for ACh-, Nic- and PC-mediated inhibition of IL-1β release. Furthermore, we hypothesize that LPC and G-PC, both PC-bearing metabolites of phosphatidylcholines, can function as agonists of nAChRs in monocytes and contribute to the regulation of IL-1β release. In addition, we characterize, which nAChR subunits are required for the inhibitory effect mediated by LPC and G-PC. Finally, we investigate if LPC, G-PC and PC induce ionotropic functions at heterologously expressed canonical nAChRs.

## Materials and Methods

### Animals

This study was carried out in accordance with the recommendations of the NIH “Guide for the Care and Use of Laboratory Animals”. The protocols for animal experiments were approved by the Regierungspräsidium Giessen, Hesse, Germany (permit number 549_M; R.P. Nr. Gi 20/23-Nr. A12/2011; R.P. Nr. Gi 20/23-Nr. A10/2011). Adult female *Xenopus laevis* (Xenopus-Express, Le Bourg, France) as well as male and female wild-type (WT) and *Chrna7* (C57B1/6J), *Chrna9* (129S-*Chrna9*^tm1Bedv/^J) and *Chrna10* (129S4-*Chrna10*^tm1Bedv/^Mmucd) gene-deficient mice were used. The generation and characterization of every gene-deficient mouse strain was described before (Orr-Urtreger et al., 1997; Vetter et al., 1999, 2007; Whiteaker et al., 2007). *Chrna 7* gene-deficient mice were obtained from the Jackson Laboratories after being re-derived, whereas *Chrna 9* and *Chrna 10* gene-deficient mice were kindly provided by Prof. Douglas E. Vetter. The genotype of every mouse was evaluated by PCR. The corresponding WT background strain for every gene-deficient mouse was used as a control.

### Mononuclear Leukocytes from Mice

Mouse peripheral blood mononuclear cells (PBMCs) were freshly isolated from heparinized blood obtained from WT, *Chrna7*, *Chrna9*, or *Chrna10* gene-deficient mice using Percoll (GE Healthcare Bio-Sciences AB, Uppsala, Sweden; 1.082 g/ml) density gradient centrifugation. Mononuclear cells were cultured for 2 h in 24-well-plates at 37°C, 5% CO_2_, in RPMI 1640 (Gibco/Life Technologies, Carlsbad, CA, USA) supplemented with 10% fetal bovine serum (FBS Superior EU, Biochrom GmbH, Berlin, Germany) and 2 mM L-glutamine (GlutaMAX™, Gibco/Life Technologies). Thereafter, cells were stimulated with 2′(3′)-O-(4-benzoylbenzoyl)adenosine 5′-triphosphate trieethylammonium salt (BzATP, 100 μM, Sigma-Aldrich, Taufkirchen, Germany) for 30 min, in the presence or absence of ACh chloride (10 μM), PC chloride calcium salt tetrahydrate (PC, 100 μM) or nicotine (Nic, 100 μM; all purchased from Sigma-Aldrich, Taufkirchen, Germany). Cell culture supernatants were collected and stored at −20°C until IL-1β and lactate dehydrogenase (LDH) measurement.

### U937 Cells

The human histocytic lymphoma cell line U937 was obtained from the German Collection of Microorganisms and Cell Cultures (Braunschweig, Germany) and cultured according to the protocol described for mouse PBMCs. Cells in the log-phase of growth were transferred to 24-well plates (1 × 10^6^ cells/ml and per well) and primed with 1 μg/ml LPS from *Escherichia coli* (L2654; Sigma-Aldrich) for 5 h. Thereafter, BzATP (100 μM) was applied for 30 min, in the presence or absence of ACh (10 μM), Nic (100 μM), PC (100 μM), L-α-G-PC (Sigma-Aldrich; 0.1 μM to 1000 μM) or 1-palmitoyl-*sn*-glycero-3-phosphocholine LPC (C16:0), Sigma-Aldrich; 0.1 μM to 100 μM). To test the involvement of different subunits of nAChR, the following antagonists were applied: mecamylamine hydrochloride (Mec, 100 μM, Sigma-Aldrich), α-bungarotoxin (α-Bun, 1 μM, Tocris Bioscience, Bristol, UK), strychnine hydrochloride (Stry, 10 μM, Sigma-Aldrich) or conotoxins ArIB [V11L, V16D] (500 nM) or RgIA4 (200 nM; Ramarao and Cohen, 1998; Whiteaker et al., 2007; Innocent et al., 2008; Richter et al., 2016; Romero et al., 2017).

### Knock-Down of α7, α9 or α10 nAChR Subunit Expression

The expression of human nAChR subunits α7, α9 or α10 was attenuated using siRNA technology. Cells were transfected with ON-TARGETplus human *CHRNA7* (α7), *CHRNA9* (α9) or *CHRNA10* (α10) SMARTpool siRNA (Thermo Fisher Scientific, Schwerte, Germany) or with ON-TARGETplus non-targeting control pool (Thermo Fisher Scientific) as a negative control. Cells were transfected with 30 pmol siRNA/1 × 10^6^ cells using the Amaxa Cell Line Nucleofector Kit C and the Nucleofector II Device (both from Lonza, Cologne, Germany) according to the manufacturer’s instructions. The siRNA-mediated down-regulation of target genes was assessed 48 h after transfection by real-time RT-PCR.

### RNA Isolation and cDNA Synthesis

Total RNA was isolated from 1 × 10^6^ U937 cells using the RNesay Plus Mini Kit (Qiagen, Hilden, Germany) according to the manufacturer’s instructions. One microgram of RNA was reversely transcribed using M-MLV H^−^ Reverse Transcriptase and 1 μg of random hexamer primers (Promega, Mannheim, Germany).

### Real-Time RT-PCR

To confirm efficient and selective knock-down of nAChR subunits α7, α9 or α10 targeted selectively with siRNA, real-time RT-PCR was performed (*n* = 4 per experimental group, each sample was assessed in duplicates) using the ABI 7900 Sequence Detection System (Applied Biosystems, Foster City, CA, USA) and Platinum SYBER green qPCR Super Mix-UDG (Invitrogen, Karlsruhe, Germany). The hydroxymethylbilane synthase (HMBS) gene was selected as a reference gene, as it was reported not to be regulated in monocytes under various culture conditions and stimulations (Moosig et al., 2006). Primers specific for the detection of *HMBS*, α7, α9 and α10 nAChR subunits were synthesized by MWG Biotech (Ebersberg, Germany) and their sequences have been published before (Hecker et al., 2015). Changes in the mRNA expression levels of nAChR were calculated by the 2^∆CT^ method, where ∆CT represents the difference between the CT value of *HMBS* gene and the CT value of gene of interest. The mean of the mRNA expression values from cells transfected with non-targeting (scrambled) siRNA was set to one and the values from cells transfected with siRNA specific for nAChR were calculated accordingly.

### ELISA

The IL-1β concentration in the cell culture supernatants was determined by mouse- or human- specific Quantikine Immunoassays (R&D Systems, Mineapolis, MN, USA) according to the manufacturer’s instructions.

### LDH Measurement

The viability of the cells was estimated by measurement of LDH in cell culture supernatants by the Non-Radioactive Cytotoxicity Assay (Promega, Madison, WI, USA) according to the manufacturer’s instructions. To assess the proportion of dead cells, a maximum LDH release control was generated. For this purpose, an equivalent number of U937 cells were disrupted by freezing them twice at −80°C. The LDH value of the sample of interest was compared with the total content of LDH in the control sample, which was set to 100% and the relative cell death was calculated accordingly.

### Isolation and Culture of Oocytes

*Xenopus laevis* oocytes were purchased from Ecocyte Bioscience (Castrop-Rauxel, Germany) or obtained from adult female frogs (Xenopus-Express, Le Bourg, France) and prepared as previously described (Richter et al., 2016). Defolliculated oocytes were stored in oocyte Ringer’s solution (ORi) containing (in mM) 90 NaCl, 1 KCl, 2 CaCl_2_, 5 HEPES (4-(2-hydroxyethyl)-piperazine-1-ethanesulfonic acid), 2.5 pyruvate, 20 mg/ml penicillin and 25 mg/ml streptomycin (pH 7.4) at 16°C. All chemicals used for ORi preparation were purchased from Fluka (Deisenhofen, Germany), except for HEPES, penicillin and streptomycin (Sigma-Aldrich).

### Heterologous Expression of Human nAChRs in Oocytes and Electrophysiological Whole-Cell Measurements

The cDNA clones of human α9, human α10 and human 43 kDa receptor-associated protein of the synapse (*RAPSN*) in the pTNT vector were obtained from Eurofins Genomics (Ebersberg, Germany). Capped cRNA was synthesized using an *in vitro* transcription kit (T7 RiboMAX™ Large Scale RNA Production System Kit, Promega, Mannheim, Germany). The cRNA encoding human α7 was kindly provided by G. Schmalzing (Department of Molecular Pharmacology, RWTH Aachen University, Aachen, Germany). In brief, a plasmid containing the entire coding region of human α7 (GenBank NM_ 000746.5, Addgene plasmid #62276; Wang et al., 2014) was purchased from Addgene (Teddington, UK^1^) and subcloned via gateway PCR from the pcDNA3.1 vector into a gateway-modified version of the oocyte expression vector pNKS2 (Gloor et al., 1995). The plasmid was linearized downstream of the vector-provided polyA tail with XhoI and transcribed with SP6 RNA polymerase into capped and polyadenylated cRNA, as previously described (Stolz et al., 2015).

Oocytes were injected with cRNA encoding nAChR subunit α7 (20 ng/oocyte) or with cRNA combinations for subunits α7 and α9 (20 ng/oocyte, each), or subunits α9 and α10 (20 ng/ oocyte, each), or α7, α9 and α10 (16, 19, 19 ng/oocyte, respectively). *RAPSN* (5 ng/oocyte) was co-injected in all experiments to increase the expression levels and to obtain stable nAChR expression (Froehner et al., 1990). The cRNA was dissolved in nuclease-free water and injected in a volume of 50.6 nl per oocyte. In all two-electrode voltage-clamp (TEVC) experiments representative controls were performed with oocytes that were injected with the same amount of nuclease-free water.

TEVC measurements were performed on cRNA- or water-injected oocytes (incubation time 3–5 days). The membrane voltage was clamped to −60 mV using a TEVC amplifier (Warner Instruments, Hamden, CT, USA), and transmembrane currents (*I*_M_) were low-pass filtered at 1000 Hz (Frequency Devices 902, Haverhill, MA, USA) and recorded with a strip chart recorder (Kipp and Zonen, Delft, Netherlands). Oocytes were placed in a perfusion chamber and perfused (gravity driven) with ORi without pyruvate and antibiotics (pH 7.4). In all experimental groups, measurements were performed on oocytes isolated from at least two different *Xenopus laevis* individuals.

For experiments examining the inhibition and recovery from inhibition of ACh-gated currents by LPC and G-PC, oocytes were injected with a 1:1 ratio of cRNA encoding human α9 and α10 or α7 alone and incubated at 17°C for 2–3 days prior to use. Oocytes were placed in a 30 μl chamber and continuously perfused by gravity with a solution containing (in mM) 96 NaCl, 2.5 KCl, 1.8 CaCl_2_ and 1 MgCl_2_ (pH 7.4). Stock solutions of LPC or G-PC were prepared in water and diluted with perfusion solution to a final concentration of 1 μM or 100 μM, respectively. The membranes were clamped at a holding potential of −70 mV and stimulated with 1 s pulses of 100 μM ACh once every 60 s until a steady-state baseline response was observed. Then, the perfusion solution was switched to one containing LPC or G-PC and the ACh-evoked responses were monitored for changes in amplitude and the data for inhibition of the ACh-evoked responses were normalized to three averaged control pulses, in the absence of LPC or G-PC, and analyzed with an exponential decay equation. The data for inhibition and recovery of inhibition by LPC or G-PC were best fit with single exponential equations.

### Statistical Analysis and Data Processing

Data were analyzed using SPSS software (Munich, Germany) by Wilcoxon signed-rank test or by the non-parametric Kruskal-Wallis test, followed by the Mann-Whitney rank-sum test. A *p* value below 0.05 was considered as statistically significant and marked with *. Data were visualized using program Inkscape version 0.92 (Free and Open Source Software licensed under the GPL).

## Results

### Single Gene Deletions of *Chrna7*, *Chrna9* or *Chrna10* Fully Abolish ACh-, Nic- or PC-Mediated Inhibition of BzATP-Induced IL-1β Release by Mouse PBMCs

**Figure 1:**
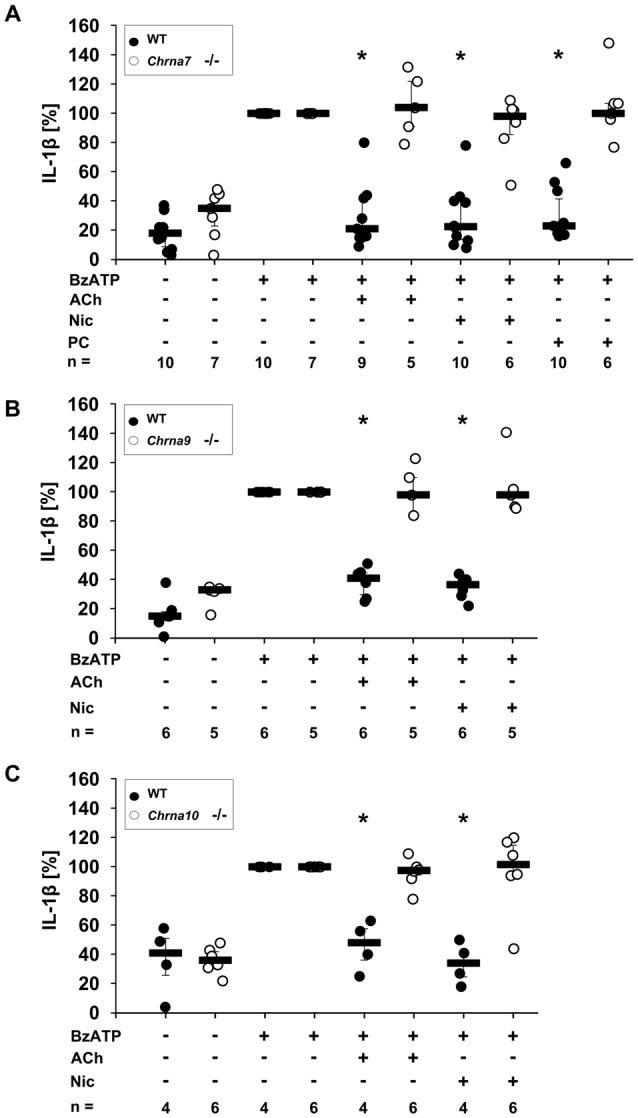
Release of interleukin-1β (IL-1β) by mouse peripheral blood mononuclear cells (PBMCs) from wild-type (WT) mice and mice deficient in *Chrna7*, *Chrna9* or *Chrna10*. Freshly isolated PBMCs were left untreated or stimulated with 2′(3′)-O-(4-benzoylbenzoyl)adenosine 5′-triphosphate triethylammonium salt (BzATP, 100 μM) for 30 min, in the presence or absence of acetylcholine (ACh, 10 μM) **(A)**, nicotine (Nic, 100 μM) **(B)** or phosphocholine (PC, 100 μM) **(C)**. BzATP induced the release of IL-1β in PBMCs obtained from all mouse strains. ACh, Nic and PC significantly inhibited the IL-1β release by PBMCs from WT mice **(A–C)**. In contrast, the IL-1β release by PBMCs from mice deficient in *Chrna7* was not affected by ACh, Nic and PC **(A)**. Similarly, a deficiency in *Chrna9* **(B)** or *Chrna10* **(C)** abolished the inhibitory effect of ACh and Nic. The IL-1β concentration was determined by ELISA. In each experiment, the IL-1β concentrations obtained after stimulation with BzATP alone were set to 100% and all other values were calculated accordingly. Data are presented as individual data points, bars represent median, whiskers percentiles 25 and 75. *p ≤ 0.05 significantly different from samples where BzATP was given alone, Wilcoxon signed-rank test.

ACh and Nic, known canonical agonists of nAChRs, as well as PC have been reported to inhibit ATP-mediated IL-1β release by human monocytes by a mechanism involving nAChRs (Hecker et al., 2015; Richter et al., 2016). To further determine the involvement of nAChR subunits α7, α9 and/or α10 in this mechanism, PBMCs from WT mice and mice deficient in single nAChR subunit genes were investigated. PBMCs isolated from WT mice or from *Chrna7*, *Chrna9* or *Chrna10* gene-deficient mice spontaneously released low amounts of IL-1β into the cell culture medium (Figures 1A–C). In response to stimulation with BzATP, an agonist of ATP receptor P2X7, the concentration of IL-1β was increased (Figures 1A–C). ACh, Nic and PC significantly inhibited the BzATP-induced release of IL-1β from WT PBMCs (Figures 1A–C, *p* ≤ 0.05, *n* ≥ 4). In sharp contrast to cells isolated from WT animals, ACh, Nic and PC did not change BzATP-induced release of IL-1β from PBMCs obtained from *Chrna7* gene-deficient mice (Figure 1A, *n* ≥ 5). Similarly, the ACh- and Nic-mediated inhibition of IL-1β release was fully abolished in PBMCs from *Chrna9* (Figure 1B, *n* ≥ 5) or *Chrna10* (Figure 1C, *n* ≥ 4) gene-deficient mice. Previously, we demonstrated that the inhibitory effect of PC is also abolished in PBMCs deficient in *Chrna9* and *Chrna10* (Richter et al., 2016). Cell death, estimated by measurement of LDH release, remained below 9% in all experiments (data not shown).

### The nAChR Subunits α7, α9 and α10 Are Necessary for ACh-, Nic- or PC-Mediated Inhibition of BzATP-Induced IL-1β Release by U937 Cells

**Figure 2:**
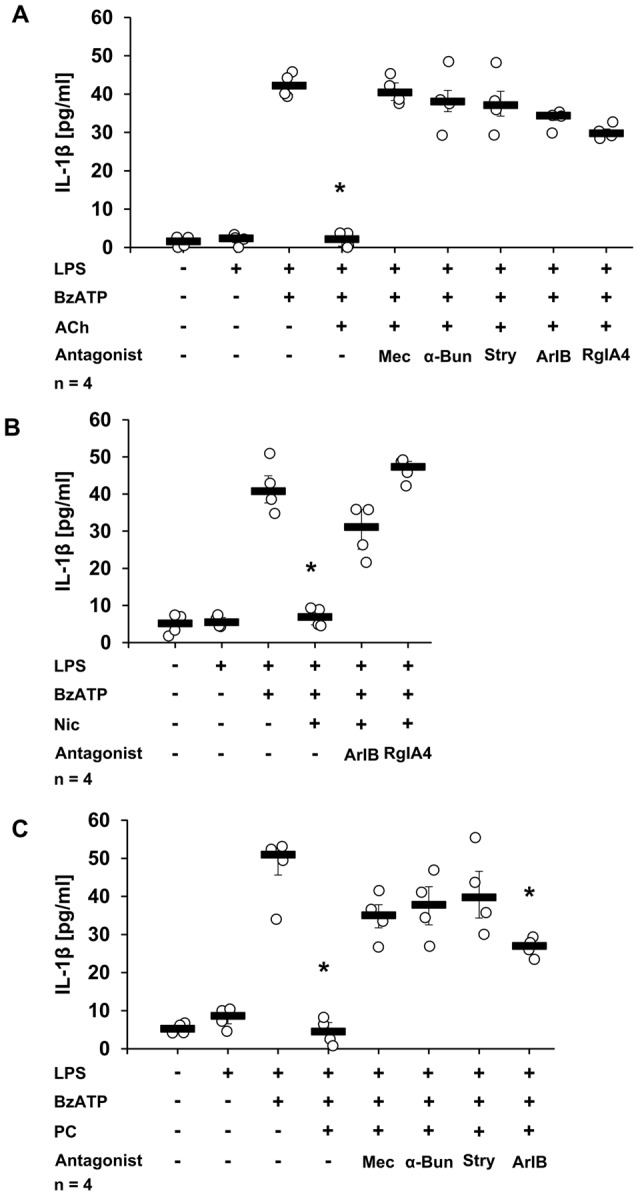
Secretion of IL-1β by U937 cells in the presence of nicotinic acetylcholine receptor (nAChR) antagonists and ACh, Nic or PC. U937 cells were primed with lipopolysaccharide (LPS; 1 μg/ml) for 5 h and BzATP (100 μM) was given for additional 30 min, in the presence or absence of ACh (10 μM) **(A)**, Nic (100 μM) **(B)** or PC (100 μM) **(C)**. BzATP induced the release of IL-1β, which was inhibited in the presence of ACh, Nic or PC **(A–C)**. The inhibitory potential of ACh **(A)** was abolished by mecamylamine (Mec, 100 μM), α-bungarotoxin (α-Bun, 1 μM), strychnine (Stry, 10 μM), ArIB [V11L, V16D] (500 nM) or RgIA4 (200 nM). Addition of ArIB or RgIA4 antagonized the effects of Nic **(B)**. Similarly the effect of PC was blunted by Mec, α-Bun, Stry and ArIB **(C)**. The IL-1β concentration was quantified by ELISA. Data were analyzed by Kruskal-Wallis test followed by Mann-Whitney rank sum test and presented as individual data points, bars represent median, whiskers percentiles 25 and 75. *p ≤ 0.05 significantly different from samples where BzATP was given alone.

To corroborate the results obtained in mouse PBMCs and to confirm that the inhibitory effects of ACh, Nic and PC depend on the activation of nAChRs composed of α7, α9 and/or α10 subunits in human monocytic cells as well, a panel of nAChR antagonists was used. As expected, ACh, Nic or PC completely inhibited the IL-1β release from human monocytic U937 cells primed with LPS and stimulated with BzATP (Figures 2A–C, *p* ≤ 0.05, *n* = 4). In the presence of the general nAChR antagonist Mec (Philip et al., 2012), the inhibitory effect of ACh and PC was lost (Figures 2A,C). Similarly, the nAChR antagonists α-Bun and Stry, that preferentially inhibit nAChRs containing α7 or α9 subunits (McIntosh et al., 2009; Kudryavtsev et al., 2015), effectively blocked the inhibitory effects of ACh and PC. As published before, Nic was also ineffective in the presence of Mec, α-Bun or Stry (Hecker et al., 2015). In line with these observations, ArIB [V11L, V16D], a selective antagonist of α7 nAChRs (Whiteaker et al., 2007; Innocent et al., 2008), abolished the inhibitory effects of ACh and Nic (Figures 2A,B), whereas the effect of PC was strongly diminished but not fully inhibited (Figure 2C). Suppression of the activity of the nAChR subunit α9 by the treatment with the selective α-conotoxin RgIA4 (Vincler et al., 2006; Romero et al., 2017) abolished the inhibitory effect mediated by ACh or Nic (Figures 2A,B). It has been shown before, that the inhibitory effect of PC was prevented in the presence of RgIA4 (Richter et al., 2016). Of note, the application of nAChR antagonists alone had no effect on the IL-1β release (Hecker et al., 2015; Richter et al., 2016).

**Figure 3:**
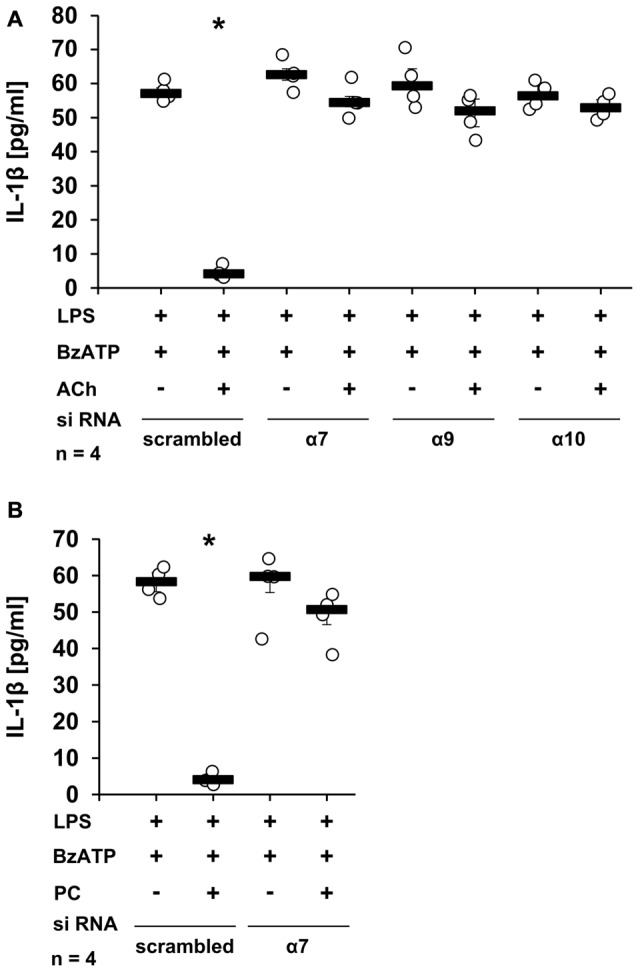
Down-regulation of nAChR subunits α7, α9 or α10 by siRNA abolished the inhibitory potential of ACh or PC. U937 cells were transfected with siRNA targeting *CHRNA7* (α7)*, CHRNA9* (α9)*, CHRNA10* (α10) or with scrambled siRNA. Forty-eight hours after transfection, cells were primed with lipopolysaccharide (LPS; 1 μg/ml) for 5 h and BzATP (100 μM) was given for additional 30 min in the presence or absence of ACh (10 μM) **(A)** or PC (100 μM) **(B)**. BzATP induced the release of IL-1β, which was inhibited in the presence of ACh or PC in cells transfected with scrambled siRNA. Down-regulation of the nAChR subunit α7 blunted the effect of ACh **(A)** or PC **(B)**. Similarly, reduced expression of the α9 and α10 nAChR subunits diminished the inhibitory potential of ACh **(A)**. The IL-1β concentration was measured by ELISA. Data were analyzed by Kruskal-Wallis test followed by Mann-Whitney rank sum test and presented as individual data points, bars represent median, whiskers percentiles 25 and 75. *p ≤ 0.05 significantly different from samples where BzATP was given alone.

To further confirm the importance of nAChR subunits α7, α9 and/or α10 in the ACh-, and PC-mediated inhibition of BzATP-induced IL-1β secretion, the expression of these subunits was down-regulated in U937 cells by siRNA. As expected, ACh and PC fully inhibited IL-1β release from U937 cells transfected with scrambled siRNA (Figures 3A,B, *p* ≤ 0.05, *n* = 4), whereas silencing the expression of the α7 subunit blunted the inhibitory potential of ACh and PC in U937 cells primed with LPS and stimulated with BzATP (Figures 3A,B). Similarly, a reduced expression of α9 or α10 subunits blocked the inhibitory mechanism mediated by ACh (Figure 3A). Of importance, gene-silencing did not impair BzATP-mediated IL-1β release (Figures 3A,B).

**Figure 4:**
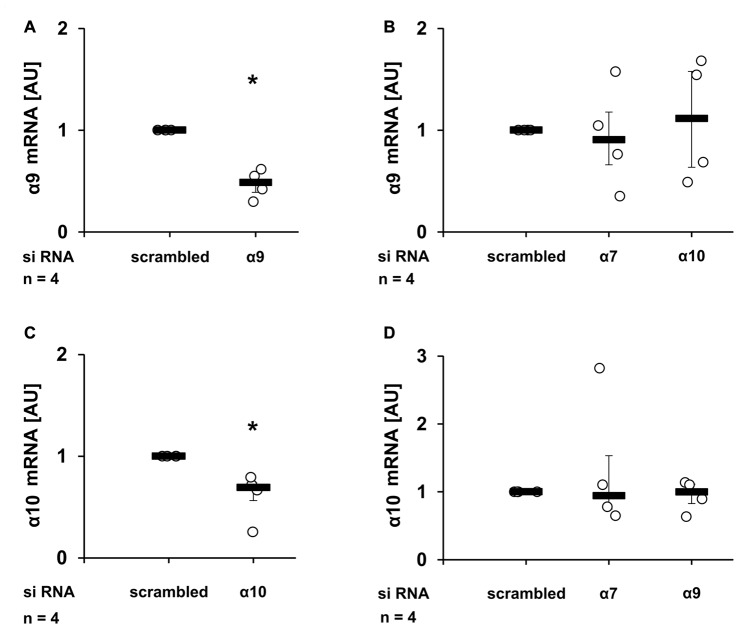
Reduction of mRNA expression of nAChR subunits α9 and α10 upon siRNA transfection. U937 cells were transfected with scrambled siRNA or with siRNA specific for *CHRNA7* (α7)*, CHRNA9* (α9) or *CHRNA10* (α10). Forty-eight hours after transfection, the mRNA expression of α9 **(A,B)** or α10 **(C,D)** was analyzed by real-time RT-PCR. Transfection efficiently **(A,C)** and selectively **(B,D)** down-regulated the mRNA expression of the gene targeted with gene-specific siRNA. Data were analyzed by Mann-Whitney rank sum test and presented as individual data points, bars represent median, whiskers percentiles 25 and 75. *p ≤ 0.05 significantly different from samples transfected with scrambled siRNA.

To control for the efficiency and specificity of subunit knock-down, the mRNA expression of nAChR subunits α9 and α10 was quantified by real-time RT-PCR (Figures 4A–D). As already reported (Hecker et al., 2015), transfection of siRNAs targeting the α9 or the α10 nAChR subunit resulted in a significant down-regulation of mRNA of subunit α9 (Figure 4A, *p* ≤ 0.05, *n* = 4) and α10 (Figure 4C, *p* ≤ 0.05, *n* = 4) respectively, when compared with cells transfected with scrambled siRNA. In contrast, transfection with siRNA targeting nAChR subunits α7 or α10 did not change the α9 mRNA expression (Figure 4B). Similarly, transfection with siRNA specific for the α7 or α9 nAChR subunits did not influence the expression of subunit α10 (Figure 4D). The mRNA expression of nAChR subunit α7 was detected at a very low level, which could not be reliably quantified. Due to the lack of specific antibodies (Moser et al., 2007; Rommel et al., 2015) it is problematic to investigate protein expression levels of nAChR subunits.

### LPC and G-PC Dose-Dependently Inhibit BzATP-Induced IL-1β Release by U937 Cells

**Figure 5:**
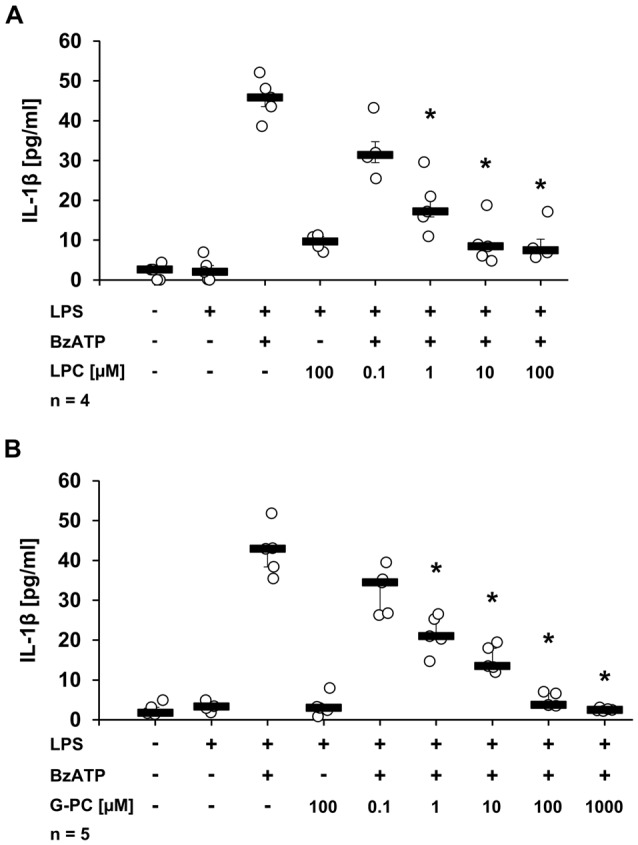
Dose-dependent inhibition of IL-1β release by lysophosphatidylcholine (LPC) or glycerophosphocholine (G-PC). U937 cells were primed with LPS (1 μg/ml) for 5 h and BzATP (100 μM) was applied for additional 30 min, in the presence or absence of different concentrations of LPC (0.1–100 μM) or G-PC (0.1–1000 μM). The BzATP-induced IL-1β release was inhibited in the presence of LPC or G-PC. The IL-1β concentration was determined by ELISA. Data were analyzed by Kruskal-Wallis test followed by Mann-Whitney rank sum test and presented as individual data points, bars represent median, whiskers percentiles 25 and 75. *p ≤ 0.05 significantly different from samples where BzATP was given alone.

To test whether other metabolites of phosphatidylcholines, besides PC, could also inhibit BzATP-induced IL-1β release in U937 cells, the inhibitory effects of LPC and G-PC were examined. LPC (IC_50_ ~1 μM; Figure 5A, *p* ≤ 0.05, *n* = 5) as well as G-PC (IC_50_ ~1 μM; Figure 5B, *p* ≤ 0.05, *n* ≥ 4) dose-dependently inhibited BzATP-induced IL-1β release without affecting the viability of the cells, as measured by LDH release (data not shown). Application of LPC (100 μM) or G-PC (100 μM) alone did not induce IL-1β release.

### The nAChR Subunits α9 and α10 Are Mandatory for LPC- or G-PC-Mediated Inhibition of BzATP-Induced IL-1β Release by U937 Cells

**Figure 6:**
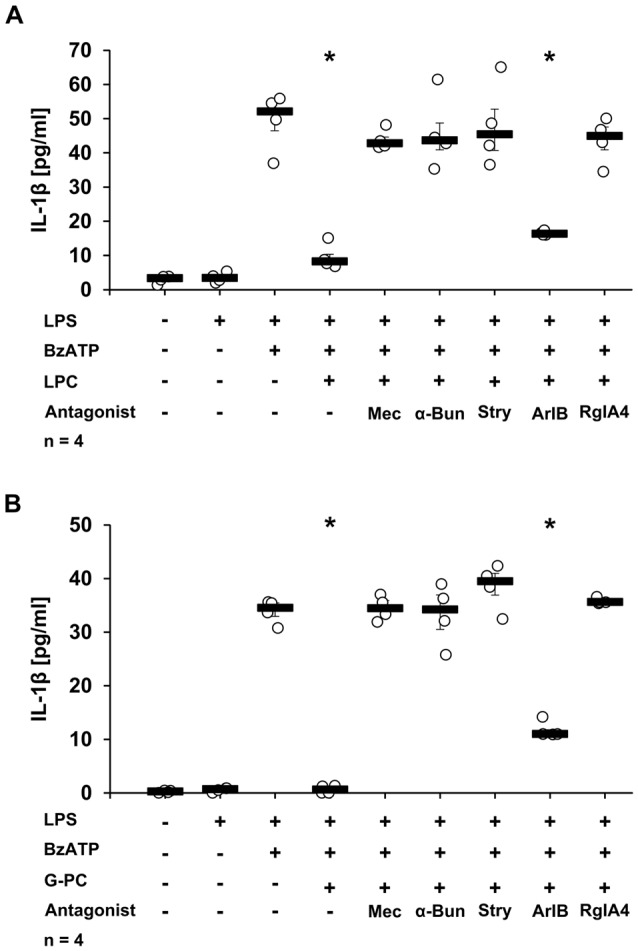
Secretion of IL-1β by U937 cells in the presence of nAChR antagonists and LPC or G-PC. U937 cells were primed with LPS (1 μg/ml) for 5 h and BzATP (100 μM) was given for additional 30 min, in the presence or absence of LPC (10 μM) **(A)** or G-PC (100 μM) **(B)**. LPC or G-PC inhibited efficiently the BzATP-mediated IL-1β release. The inhibitory potential of LPC and G-PC was abolished by mecamylamine (Mec, 100 μM), α-bungarotoxin (α-Bun, 1 μM), strychnine (Stry, 10 μM), or RgIA4 (200 nM). In contrast, addition of ArIB [V11L, V16D] (500 nM), an α7 nAChR subunit-specific antagonist, did not abolish the inhibitory effect mediated by LPC or G-PC. The IL-1β concentration was quantified by ELISA. Data were analyzed by Kruskal-Wallis test followed by Mann-Whitney rank sum test and presented as individual data points, bars represent median, whiskers percentiles 25 and 75. *p ≤ 0.05 significantly different from samples where BzATP was given alone.

**Figure 7:**
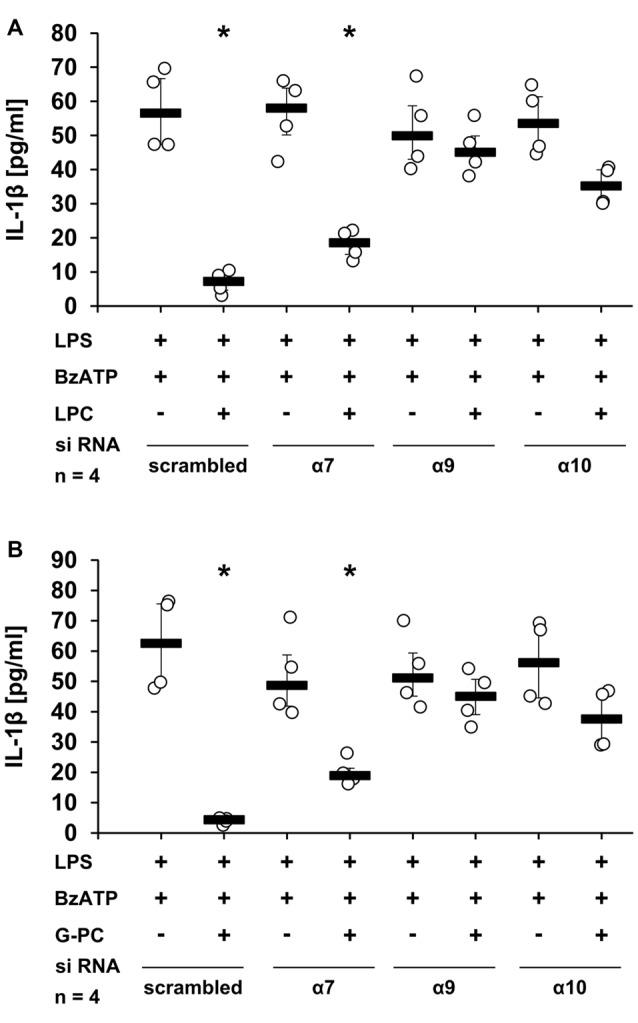
Down-regulation of nAChR subunits α9 or α10 by siRNA abolished the inhibitory potential of LPC and G-PC. U937 cells were transfected with siRNA targeting *CHRNA7* (α7)*, CHRNA9 (*α9)*, CHRNA10* (α10) or with scrambled siRNA. Forty-eight hours after transfection cells were primed with LPS (1 μg/ml) for 5 h and BzATP (100 μM) was given for additional 30 min in the presence or absence of LPC (10 μM) **(A)** and G-PC (100 μM) **(B)**. BzATP induced the release of IL-1β, which was inhibited in the presence of LPC or G-PC in cells transfected with scrambled siRNA. Reduced expression of α9 or α10 subunits abrogated the inhibitory potential of LPC and G-PC. In contrast, down-regulation of the α7 subunit expression only slightly inhibited the LPC- or G-PC-mediated mechanism. The IL-1β concentration was measured by ELISA. Data were analyzed by Kruskal-Wallis test followed by Mann-Whitney rank sum test and presented as individual data points, bars represent median, whiskers percentiles 25 and 75. *p ≤ 0.05 significantly different from samples where BzATP was given alone.

To analyze the involvement of nAChR subunits α7, α9 and/or α10 in the LPC- and G-PC-mediated inhibition of BzATP-induced IL-1β secretion, a panel of nAChR antagonists was applied. The inhibitory effects of LPC and G-PC were fully prevented in the presence of Mec, α-Bun, Stry or RgIA4. In contrast, ArIB, the nAChR α7 specific antagonist, at best slightly attenuated the inhibitory effects mediated by LPC and G-PC (Figures 6A,B, *p* ≤ 0.05, *n* = 4). To further confirm these results, the expression of nAChR subunits α7, α9 or α10 was selectively down-regulated using siRNA (Figures 7A,B, *p* ≤ 0.05, *n* = 4). Silencing of single nAChR subunits, or transfection of scrambled siRNA, did not influence the BzATP-induced IL-1β release. Importantly, silencing the expression of nAChR subunits α9 or α10 effectively abolished the inhibitory potential of LPC and G-PC. In contrast, down-regulation of the α7 nAChR subunit only slightly impaired the inhibitory effects of LPC and G-PC (Figures 7A,B, *p* ≤ 0.05, *n* = 4).

### LPC and G-PC Do Not Induce Ion Channel Functions at Heterologously Expressed nAChRs

Next, we investigated the effect of LPC and G-PC at canonical ionotropic nAChRs. For this purpose, we used *Xenopus laevis* oocytes as a heterologous expression system for different combinations of human α7, α9 and α10 nAChR subunits and performed TEVC measurements to assess ion channel functions. We demonstrated previously, that PC does not evoke ion currents at heterologously expressed homomeric α9 and heteromeric α9α10 nAChR (Richter et al., 2016). Here we tested the effects of PC at different combinations of human α7, α9 and α10 nAChR subunits. To control for the expression of nAChR in all experiments, the nAChR agonist ACh (100 μM; Papke et al., 1996; Azam and McIntosh, 2012) was used as a positive control.

**Figure 8:**
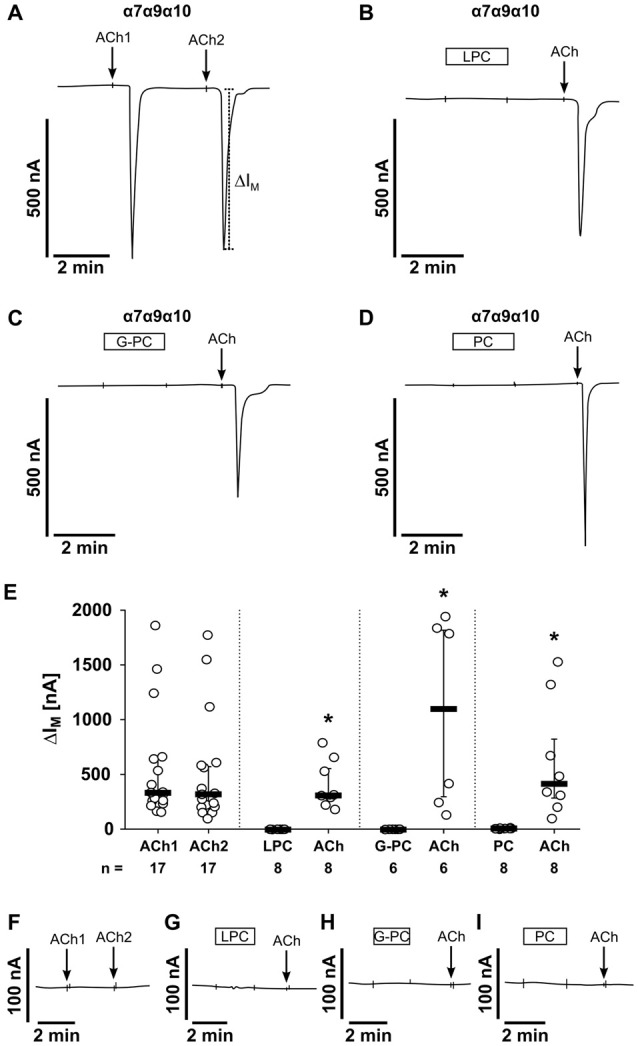
LPC, G-PC and PC do not induce ion channel functions at oocytes co-expressing nAChR subunits α7, α9 and α10. Two-electrode voltage-clamp (TEVC) measurements were performed on *Xenopus laevis* oocytes that heterologously co-expressed human α7, α9 and α10 nAChR subunits. **(A)** Representative current curve. The application of ACh (100 μM; 1 s) induced repetitive responses of transmembrane ion currents (∆*I*_M_; ACh1 and ACh2). **(B–D)** Initial application of LPC (100 μM; 2 min) **(B)**, G-PC (100 μM; 2 min) **(C)** as well as PC (100 μM; 2 min) **(D)** had no impact on ∆*I*_M_, whereas ACh induced a current response. **(E)** Graphical representation of the results of experiments as shown in **(A–D)**. **(F–I)** In water-injected control oocytes neither repeated application of ACh (*n* = 16), nor application of LPC (*n* = 6), G-PC (*n* = 4) or PC (*n* = 6) had any effect on ∆*I*_M_. All changes of ∆*I*_M_ induced by cholinergic stimulation are shown as individual data points, bars represent median, whiskers percentiles 25 and 75. Statistical analyses were performed using the Wilcoxon signed-rank test. *p ≤ 0.05 significantly different from the ∆*I*_M_ values before.

The first set of experiments was performed on oocytes that were injected with cRNA encoding for α7, α9 and α10 nAChR subunits (Figure 8). As expected, application of ACh (ACh1) evoked currents (∆ *I*_M_) that were reversible and repeatable by subsequent ACh application (ACh2; Figure 8A). The ACh-induced effects (ACh1, ACh2) were not significantly different (*n* = 17, *p* = 0.14; Figures 8A,E). Next, LPC (100 μM) was tested on oocytes expressing nAChR subunits α7, α9 and α10 (Figure 8B). While LPC (2 min) did not elicit ion currents, subsequent application of ACh resulted in significant current responses (*n* = 8, *p* = 0.001; Figures 8B,E). In the same way, G-PC (100 μM; Figure 8C) and PC (1 mM; Figure 8D) were tested. Again, neither G-PC nor PC induced ion currents, while application of ACh induced a current response (G-PC: *n* = 6, *p* = 0.001, Figures 8C,E; PC: *n* = 8, *p* = 0.001; Figures 8D,E). In water-injected control oocytes, ACh (*n* = 4), LPC (*n* = 6), G-PC (*n* = 4) and PC (*n* = 6) and had no impact on ∆*I*_M_ (Figures 8F–I).

**Figure 9:**
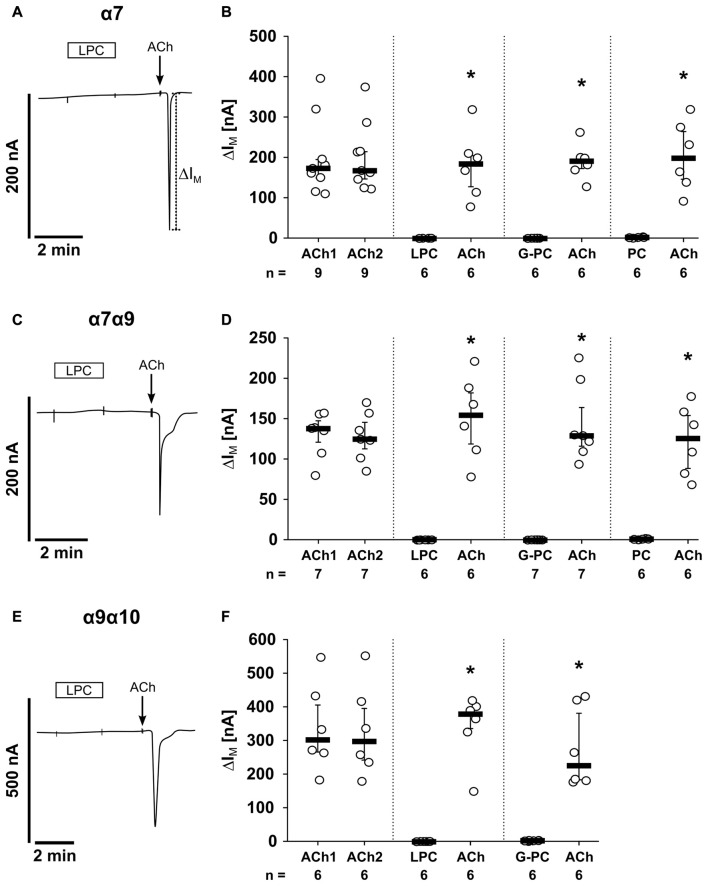
LPC, G-PC and PC do not induce ion channel activity at heterologously expressed nAChR subunits. TEVC measurements were performed on *Xenopus laevis* oocytes that expressed the human α7 nAChR subunit alone **(A,B)**, as well as co-expressed human α7 and α9 **(C,D)** or human α9 and α10 **(E,F)** nAChR subunits. In all experiments, application of ACh (100 μM; 1 s) induced repetitive stimulations of the transmembrane ion current (∆*I*_M_; ACh1 and ACh2). In contrast, initial application of LPC (100 μM; 2 min), G-PC (100 μM; 2 min) as well as PC (100 μM; 2 min) had no impact on ∆*I*_M_, whereas ACh induced a current response. All changes of ∆*I*_M_ induced by cholinergic stimulation are shown as individual data points, bars represent median, whiskers percentiles 25 and 75. Statistical analyses were performed using the Wilcoxon signed-rank test. *p ≤ 0.05 significantly different from the ∆*I*_M_ values before.

In addition, we performed experiments on oocytes that were injected with different combinations of human nAChR subunits (Figure 9). In oocytes expressing homomeric α7 nAChRs, application of LPC, G-PC or PC had no impact on ∆*I*_M_, while ACh induced significant current responses (Figures 9A,B; LPC: *n* = 6, *p* = 0.001; G-PC: *n* = 6, *p* = 0.001; PC: *n* = 6, *p* = 0.001). The same results were obtained in experiments on oocytes co-expressing nAChR subunits α7 and α9 (LPC: *n* = 6, *p* = 0.001; G-PC: *n* = 7, *p* = 0.001; PC: *n* = 6, *p* = 0.001; Figures 9C,D). It was previously shown, that PC does not induce ion channel functions on heterologously expressed heteromeric α9α10 nAChR (Richter et al., 2016). Here, we investigated the impact of LPC and G-PC on heteromeric α9α10 nAChR (Figures 9E,F). Again, application of LPC and G-PC had no effect on ∆*I*_M_, while ACh induced a significant current response (Figures 9E,F; LPC: *n* = 6, *p* = 0.001; G-PC: *n* = 6, *p* = 0.001). In all experiments the ACh-induced effect was repeatable (ACh1, ACh2) and not significantly different compared to the first response (α7: *n* = 9, *p* = 0.432; α7α9: *n* = 7, *p* = 0.645; α9α10: *n* = 6, *p* = 0.65; Figures 9B,D,F). LPC, G-PC, PC and ACh did not induce changes in ion currents of water-injected control oocytes (data not shown).

### Inhibition of ACh-Induced Currents by LPC and G-PC

**Figure 10:**
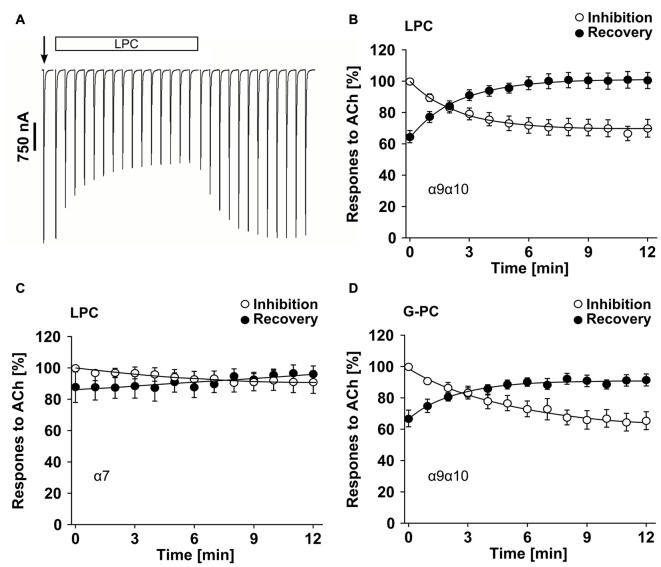
LPC and G-PC desensitize ACh-gated currents mediated by nAChRs heterologously expressed in *Xenopus laevis* oocytes. TEVC experiments were performed on oocytes that expressed human homomeric α7 or heteromeric α9α10 nAChRs. **(A)** Representative traces of ACh-gated currents mediated by α9α10 nAChRs illustrating the inhibitory effect of LPC (1 μM). The current traces represent 30 s recordings each and are shown concatenated omitting the 30 s gap between each individual trace. The oocytes were continuously perfused with control solution and stimulated with 1 s pulses of ACh (100 μM) once per min until steady-state baseline responses were observed (indicated by the arrow). Subsequently, the control solution was switched to one containing LPC and the ACh-gated currents were monitored for changes in amplitude for 12 min. Thereafter, the perfusion solution was switched back to control and the ACh-gated currents were monitored for recovery. **(B)** Graphical representation of the experimental results shown in **(A)** (*n* = 12). **(C)** Similar experiments as those in **(A,B)** were performed on α7 nAChR expressing oocytes to assess the effects of LPC on ACh-gated currents mediated by this nAChR subtype (*n* = 6). **(D)** Analysis of inhibition and recovery from inhibition of ACh-gated currents by G-PC (100 μM) on α9α10 nAChR expressing oocytes (*n* = 6). The error bars in all graphs denote the standard error of mean (SEM) from the indicated numbers of oocytes. The data in **(B–D)** were analyzed with single exponential equations and the *X-axes* represent the temporal duration of each experimental condition (absence or presence of LPC or G-PC).

Previously, we demonstrated that PC blunts classical ion channel responses at α9α10 nAChR heterologously expressed by *Xenopus laevis* oocytes (Richter et al., 2016). To test if LPC and G-PC have an impact on ACh-induced ion currents of nAChR, we performed TEVC measurements and monitored current responses induced by ACh (100 μM, 1 s pulses) in presence or absence of LPC or G-PC (Figure 10). In experiments on oocytes expressing heteromeric α9α10 nAChR, LPC (1 μM) decreased the ACh-evoked responses to 69 ± 2% (*n* = 12) of control values after a 12 min exposure to the compound (Figures 10A,B). Thereafter, LPC was washed out and the current responses recovered to 101 ± 2% (*n* = 12) after 12 min (Figures 10A,B). Similar experiments were performed on oocytes expressing homomeric α7 nAChR (Figure 10C). In presence of LPC, the ACh-evoked currents were reduced to 89 ± 7% (*n* = 6) of control values after a 12 min perfusion (Figure 10C). The responses recovered to 101 ± 6% (*n* = 6) of control values after a 12 min wash-out period (Figure 10C). In oocytes that expressed heteromeric α9α10 nAChR, G-PC (100 μM) decreased the ACh-induced currents to 60 ± 7% (*n* = 6) of control values (Figure 10D). After a 12 min wash-out period of G-PC, the ACh-induced responses recovered to 90 ± 2% (*n* = 6) of control values (Figure 10D).

## Discussion

Anti-inflammatory effects of nAChR agonists in macrophages were repeatedly described and the term “anti-inflammatory cholinergic reflex” was coined more than a decade ago. It was suggested that ACh of vagal origin activates nAChR composed of subunit α7 and controls the synthesis of pro-IL-1β and other pro-inflammatory cytokines on the transcriptional and post-transcriptional level (Borovikova et al., 2000; de Jonge et al., 2005; Rosas-Ballina et al., 2011; Olofsson et al., 2012). We recently discovered that Nic, Cho and ACh efficiently reduce the ATP-induced inflammasome activation and IL-1β maturation in human monocytes. Ligand-binding to monocytic nAChRs containing subunits α7, α9 and/or α10 triggers a metabotropic response that efficiently inhibits ATP-signaling at P2X7 receptor (Hecker et al., 2015). Similar cholinergic effects on monocytes are also provoked by PC, PC-modified macromolecules and DPPC (Hecker et al., 2015; Richter et al., 2016; Backhaus et al., 2017). In the current study: (i) we demonstrate that LPC and G-PC, common metabolites of phosphatidylcholines, also activate monocytic nAChR; (ii) we determine the nAChR subunit requirements of different conventional and non-conventional nicotinic agonists; and (iii) we provide evidence that LPC, G-PC and PC do not evoke ion currents at heterologously expressed nAChR, but rather act as silent agonists.

We provide evidence that LPC and G-PC function as nAChR agonists that dose-dependently and efficiently inhibit the BzATP-mediated IL-1β release by LPS-primed human monocytic U937 cells. Interestingly, the IC_50_ of both compounds is one order of magnitude lower than that of PC (Hecker et al., 2015) or DPPC (Backhaus et al., 2017). Importantly, effective concentrations of LPC, G-PC and PC can be detected in human plasma (Ilcol et al., 2002, 2005; Drobnik et al., 2003; Quehenberger et al., 2010). As LPC and G-PC are metabolites of phosphatidylcholines, different cell signaling pathways involving phospholipases as well as exposure to insect or snake venoms rich in phospholipases (Harris and Scott-Davey, 2013) may trigger this anti-inflammatory pathway. There are some conflicting data on immune-modulatory functions of LPC (Liu-Wu et al., 1998; Kabarowski et al., 2002; Stock et al., 2006; Carneiro et al., 2013), but none of these studies suggested a cholinergic mechanism. Anti-inflammatory effects of LPC, including a reduction of peritoneal IL-1β levels, were reported for experimental sepsis in the mouse and these effects seemed to depend on the presence of 18 carbon molecules in the acyl chain (Yan et al., 2004), whereas we used LPC with a chain length of 16 in our experiments. Other studies suggested an induction of pro-IL-1β in human monocytes during long-term incubation with LPC with a carboxyl chain length of 16 and 18 (Liu-Wu et al., 1998) and a LPC- and Ca^2+^-dependent induction of IL-1β in LPS-primed microglial cells (Stock et al., 2006). These conflicting results might be explained by the different experimental protocols, the different cell types investigated and by the potential presence of pyrogenic contaminations that induce the expression of IL-1β.

In order to define which nAChR subunits are required for the inhibitory function of ACh, Nic and PC on the BzATP-driven release of IL-1β, we used gene-deficient mice, a panel of nAChR antagonists and performed gene-silencing experiments. We knew from previous pharmacological and gene-silencing studies that the effect of Nic is mediated via nAChR containing subunits α7, α9 and/or 10 (Hecker et al., 2015). It remained, however, unclear, if all three receptor subunits are mandatory or if they exert redundant functions. Regarding the signaling of PC, experiments on gene-deficient mice as well as pharmacological and gene-silencing studies revealed that nAChR subunits α9 and α10 are mandatory (Richter et al., 2016) but in this case the role of subunit α7 was still unclear and nothing was known about the nAChR subunit requirements for the ACh mediated effects. First, we investigated PBMCs from WT mice and mice deficient in the genes for nAChR subunits α7, α9 or α10. The results of these experiments suggested that all three receptor subunits are mandatory for the inhibition of IL-1β release by ACh, Nic and PC. In the same line, Mec, a general nicotinic antagonist (Philip et al., 2012) as well as α-Bun and Stry, antagonists of nAChR containing subunits α7 and α9 (McIntosh et al., 2009; Kudryavtsev et al., 2015), efficiently reverted the inhibitory effects of ACh, Nic (Hecker et al., 2015) and PC in LPS-primed human U937 cells. Similarly, RgIA4, a selective antagonist of nAChR subunit α9 (Vincler et al., 2006; Romero et al., 2017) and ArIB, a selective antagonist of nAChR subunit α7 (Innocent et al., 2008), fully abolished the inhibitory effect mediated by ACh, Nic and PC (Richter et al., 2016). These data were further corroborated by siRNA experiments performed here and in previous studies (Hecker et al., 2015; Richter et al., 2016). We conclude from these data that all three nAChR subunits α7, α9 and α10 are needed for signaling of ACh, Nic and PC in monocytic cells (Table 1).

**Table 1:** Concentration of agonists causing 50% inhibition (IC_50_ of the adenosine triphosphate (ATP)-mediated release of interleukin-1β (IL-1β) in monocytes and the nAChR composition needed for the signaling.

Endogenous agonist	Approximate IC_50_	Required nAChR subunits
**ACh**	10 μM ^(Hecker et al., 2015)^	α7, α9, α10
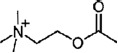
**PC**	10 μM ^(Hecker et al., 2015)^	α7, α9, α10
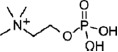
**LPC**	1 μM	α9, α10
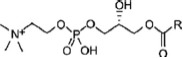
**G-PC**	1 μM	α9, α10
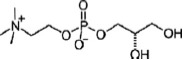
**DPPC**	10 μM ^(Backhaus et al., 2017)^	α9 and α7 or α10
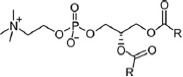

*ACh, acetylcholine; DPPC, dipalmitoylphosphatidylcholine; G-PC, glycero- phosphocholine; LPC, lysophosphatidylcholine; nAChR, nicotinic ACh receptor; R, fatty acid residue C16:0*.

Next, we investigated the receptor requirements for the more complex compounds LPC and G-PC by using both nAChR antagonists and gene-silencing of nAChR subunits by siRNA in human U937 cells. In contrast to ACh, Nic and PC, we provide evidence that only nAChR subunits α9 and α10 are mandatory for the inhibitory mechanism mediated by LPC and G-PC. There might be a biologically insignificant contribution of subunit α7 because both gene silencing und ArIB seemed to provoke a subtle impairment of the inhibitory effects of LPC and G-PC. This discrepancy might be explained by differences in the structure and the physicochemical properties among these compounds (Table 1). Interestingly, the recently reported inhibitory effects of DPPC on ATP-driven inflammasome activation also strictly depended on the α9 subunit, but in contrast to LPC and G-PC, the α10 subunit is not mandatory but it can be functionally replaced by subunit α7 (Backhaus et al., 2017).

With all due caution, we propose that the α9 nAChR subunit is the critical subunit involved in the metabotropic regulation of inflammasome activation, irrespective of the structure of the nicotinic agonist. The α9 nAChR subunit interacts with α7 and/or α10 subunits depending on the complexity of the nAChR agonist. Small agonists such as ACh or PC need all three subunits α7, α9 and α10 for signaling, the agonists LPC and G-PC depend on α9 and α10, without an essential contribution of subunit α7 (Table 1). The more bulky and hydrophobic phosphatidylcholines like DPPC, signal alternatively via subunit combination α7α9 or via α9α10 (Backhaus et al., 2017; Table 1). We have to admit that although this generalization is tempting, we only tested LPC (16:0) and DPPC up to now, but different variants of LPC and phosphatidylcholines have to be investigated before establishing a principle, and these are many and varied.

The following experiments were designed to clarify if LPC, G-PC and PC interact with canonical nAChRs that function as ligand-gated ion channels. We have previously shown that PC does not evoke ion currents in *Xenopus laevis* oocytes expressing human α9 or α9α10 nAChRs. Here, we investigated the short-term effect of LPC, G-PC and PC on ionotropic nAChR functions using *Xenopus laevis* oocytes as a heterologous expression system for human α7 nAChR as well as the expression of combinations of α7, α9 and α10 subunits. Due to the lack of specific antibodies to the nAChR investigated (Moser et al., 2007; Rommel et al., 2015), we were not able to test if all nAChR subunits were co-expressed in our experimental setup, but we were able to demonstrate that ACh induced characteristic ion currents in oocytes injected with cDNA for nAChR but not in control oocytes. In addition, we do not know which homomeric or heteromeric nAChR species form in co-injected oocytes. Of note, LPC, G-PC or PC did not induce ion currents in any of the experimental settings, wheras the classical nAChR agonist ACh did.

Although PC does not induce ion currents at classical nAChR, it seems to function as a silent agonist that desensitizes α9α10 nAChR (Richter et al., 2016). Hence, we investigated the long-term effects of LPC and G-PC on heterologously expressed nAChR. While the brief application of 100 μM LPC did not evoke responses in oocytes expressing nAChRs, application of LPC (100 μM) induced a non-specific increase of the baseline holding current during continuous perfusion of the compound. Such effects have been described before and might be due to a detergent-like action of LPC (Ikeuchi et al., 1997). Therefore, we lowered the LPC concentration in the long-term experiments to 1 μM, a concentration that still inhibited the ATP-induced IL-1β release in U937 cells.

In the inhibition and recovery from inhibition measurements, we found that in human α9α10 nAChR-expressing oocytes, ACh-mediated current responses were blunted in the presence of LPC and G-PC. The slow kinetics of inhibition and recovery from inhibition by LPC and G-PC suggest that both compounds might also function as silent agonists of the heteromeric α9α10 nAChR. However, we cannot formally exclude that LPC and G-PC act as partial antagonists at these nAChR. Interestingly, while LPC decreased the ACh-evoked current responses to 69 ± 2% in heteromeric α9α10 nAChR expressing oocytes, the ACh-evoked currents in homomeric α7 nAChR expressing oocytes were decreased only to 89 ± 7%. These findings are in accordance with our observation on the monocytic U937 cells, in which the immuno-modulatory function of LPC requires the expression of nAChR subunits α9 and α10 but not α7. In summary, LPC, G-PC and PC are potent agonists of non-classical nicotinic receptors of monocytes that provoke metabotropic functions. LPC, G-PC and PC do not induce ion currents, but they seem to act as silent agonists.

Receptor subunits α7, α9 and α10 belong to a unique class of evolutionary conserved nAChR, which in contrast to all other nAChR can form pentameric homomers (Franchini and Elgoyhen, 2006; Lipovsek et al., 2012, 2014). Homomeric α7 and α9 nAChR were also described (Elgoyhen et al., 1994), whereas the human α10 subunit alone does not form functional receptors (Elgoyhen et al., 2001). The α9 and α10 nAChR subunits can co-assemble to heteromers with pharmacological properties different from α9 homomers (Boffi et al., 2017). Heteromers of α7 and α10 have been evidenced in rat sympathetic neurons (Lips et al., 2006). Furthermore a complex interaction of nAChR subunits α7, α9 or α10 in regulating vestibular afferent gain was shown (Morley et al., 2017). In a mast cell line, interaction of nAChR subunits α7, α9 and α10 was described in the context of nicotinic inhibition of FcεRI-induced leukotriene and cytokine production (Mishra et al., 2010). However, exceedingly low nanomolar concentrations of Nic were effective in this mast cell line, which is not the case for the inhibition of ATP-induced release of IL-1β by human U937 cells (Hecker et al., 2015). In all leukocyte subtypes tested so far, nAChR subunits do not form conventional ligand-gated ion channels, but rather exert metabotropic function (Peng et al., 2004; Razani-Boroujerdi et al., 2007; Hecker et al., 2009, 2015; Mishra et al., 2010; Richter et al., 2016; Valbuena and Lerma, 2016). We published before, that Nic, Cho, PC and DPPC exert their immuno-modulatory functions via metabotropic inhibition of P2X7 receptor function (Hecker et al., 2015; Richter et al., 2016; Backhaus et al., 2017). However, the structure of non-canonical nAChR receptors exerting metabotropic functions is largely unknown; it is even unclear, if leukocytic nAChR form multimers.

There is increasing evidence, that signaling via nAChR subunit α7 induces Ca^2+^ signals and that signaling-related proteins including G proteins can interact with a cytoplasmic loop of this subunit (Kabbani et al., 2013; King and Kabbani, 2016; Valbuena and Lerma, 2016). Similar protein-protein interactions might also occur for subunit α9. In addition, direct interactions of nAChR containing subunit α6 with purinergic receptors P2X2 and P2X3 have been reported (Limapichat et al., 2014; Wieskopf et al., 2015). This interesting and emerging field of metabotropic nicotinic signaling certainly deserves more scientific attention.

Excessive release of IL-1β into the circulation in response to trauma-associated extracellular ATP is useless and dangerous, because the cytokine is swept away from the site of injury and can cause severe life-threatening SIRS and multi-organ damage (Stoecklein et al., 2012; Lord et al., 2014). As monocytes are the major source of IL-1β within the circulation (Arango Duque and Descoteaux, 2014), the inhibitory mechanisms described in this study might be of high clinical relevance. ATP-driven inflammasome activation is typical for the undesired sterile inflammation that causes a high morbidity and mortality worldwide, whereas several ATP-independent pathways lead to the desired inflammasome activation needed for defense against infection (Cauwels et al., 2014). Of note, nAChR subunit α9 plays a central role in the control of ATP-driven inflammasome activation in monocytes, whereas subunit α7 controls the expression of pro-inflammatory cytokines more generally (Borovikova et al., 2000). The specific α9-dependend pathway seems to be an interesting target that might enable the prevention of SIRS caused by multiple traumata and major surgery without inhibiting host defense against infections. As we demonstrated that the cholinergic inhibition of ATP-driven IL-1β release is also active in mice, this concept can and should be evaluated experimentally *in vivo*.

This study has several limitations and more research is needed to further corroborate our concept. One major technical issue is the lack of specific antibodies to the nAChR subunits under investigation that prevents an unequivocal proof of the specificity and efficiency of gene-silencing as well as the proof of the heterologous co-expression of nAChR subunits. At present, we cannot conclude that all LPC species act in the same way like LPC (16:0) and the same holds true for phosphatidylcholines, where only DPPC was investigated previously (Backhaus et al., 2017). It also remains to be tested, if LPC and G-PC stimulate ion currents in monocytic cells, although this is improbable as PC and DPPC do not (Richter et al., 2016; Backhaus et al., 2017). Additionally, more research is needed to identify the signaling pathways involved in the inhibitory mechanism mediated by LPC, G-PC and PC. Finally, we can only speculate on the relevance of our findings *in vivo*.

In conclusion, we demonstrated that canonical nAChR agonists as well as PC inhibit ATP-induced release of IL-1β by human and mouse mononuclear cells via nAChR subunits α7, α9 and α10. In contrast, LPC (16:0) and G-PC, more complex metabolites of phosphatidylcholines, elicit the inhibitory mechanism in human monocytes via interaction of the two nAChR subunits α9 and α10. Independent of the complexity of the compound, neither LPC, G-PC nor PC induce ionotropic functions at heterologously expressed nAChR, suggesting that PC-bearing molecules in general regulate immuno-modulatory functions without inducing canonical ion channel function at nAChR. This selective activity opens the opportunity for the development of novel anti-inflammatory therapies without inducing ionotropic functions of nAChRs.

## Author Contributions

AZ and KR participated in research design, performance of experiments, interpretation of the data and writing of the manuscript. AA, SW, KS, BF and AJH: performance of experiments, interpretation of the data and editing of the manuscript. GK-C, MA and WP participated in research design, interpretation of the data and editing of the manuscript. JMM and VG participated in research design, interpretation of the data and writing of the manuscript.

## Conflict of Interest Statement

Certain conotoxins, including RgIA4 have been patented by the University of Utah; JMM is an inventor on these patents. The other authors declare that the research was conducted in the absence of any commercial or financial relationships that could be construed as a potential conflict of interest.

## Footnotes

^1^https://www.addgene.org/62276/
